# Resistance gene profiling of beta-lactamase and carbapenemase in gram-negative blood isolates: A tertiary care hospital

**DOI:** 10.1371/journal.pone.0344856

**Published:** 2026-03-30

**Authors:** Daniel Beshah, Tesfaye Sisay Tessema, Gurja Belay Woldemichael, Esmael Besufikad Belachew, Dawit Hailu Alemayehu, Abebech Tesfaye, Dessalegn Abeje Tefera, Solomon Gizaw Derese, Tizazu Zenebe Zelelie, Abera Admasie, Tamrat Abebe, Adey Feleke Desta

**Affiliations:** 1 Department of Medical Laboratory Science, School of Biomedical and Laboratory Science, College of Health Sciences, Addis Ababa University, Addis Ababa, Ethiopia; 2 Institute of Biotechnology, Addis Ababa University, Addis Ababa, Ethiopia; 3 Department of Microbial, Cellular, and Molecular Biology, College of Natural and Computational Sciences, Addis Ababa University, Addis Ababa, Ethiopia; 4 Armauer Hansen Research Institute, Addis Ababa, Ethiopia; 5 Department of Microbiology, Immunology, and Parasitology, School of Medicine, College of Health Sciences, Addis Ababa University, Addis Ababa, Ethiopia; 6 Department of Medical Laboratory Science, Debre Berhan University, Debre Berhan, Ethiopia; 7 Department of Biology (Biotechnology program), College of Natural and Computational Sciences, Arba Minch University, Arba Minch, Ethiopia; Ahvaz Jondishapour University of Medical Sciences Faculty of Medicine, IRAN, ISLAMIC REPUBLIC OF

## Abstract

The global rise in antimicrobial resistance is largely driven by DNA-encoded, antibiotic-hydrolyzing enzymes. This study aimed to determine the prevalence of ESBL, AmpC-BL, MBL, and carbapenemase genes. A cross-sectional study was conducted from September 2018 to March 2019 using standard microbiological and molecular methods. Among 231 Gram-negative bacterial isolates, 176 (76.2%) carried beta-lactamase genes confirmed by PCR. ESBLs were detected in 117 (50.6%), MBLs in 50 (21.6%), carbapenemases in 46 (19.9%), and AmpC-BLs in 22 (9.5%). The most frequent carriers were *Klebsiella pneumoniae*, *Escherichia coli*, and *Acinetobacter species*. The dominant ESBL genes were *blaCTX-M* (85.2%) and *blaTEM* (79.5%), with *blaBEL* (17.2%) surpassing *blaSHV* (9.8%), indicating a shift in resistance patterns, while among AmpC genes, *blaFOX* (39.4%) and *blaCITM* (36.4%) were the most prevalent. The *blaOXA-23* (40.6%) and *blaKPC* (14.5%) were the most common carbapenemase genes, while *blaNDM* (39.1%) and *blaVIM* (34.8%) dominated among MBLs. The high prevalence of *blaOXA-23* in Ethiopia is a significant finding, which I have not found evidence of in similar African or Asian settings. A comparable trend in Taiwan suggests medical tourism may contribute to the spread of resistant bacteria. These findings highlight the critical importance of continuous molecular surveillance and indicate that factors like medical tourism may contribute to the international dissemination of resistant strains.

## Introduction

Antibiotic-resistant gram-negative bacteria (GNB) due to beta-lactamases and carbapenemases are a major cause of morbidity, mortality, and significant economic costs worldwide, and their incidence and spread are on the rise [1–3]. These antibiotic resistance determinants are passed from one bacterial community to another through horizontal gene transfer mechanisms [1,4]. The beta-lactamase-mediated resistance is increasingly associated with Plasmid-Encoded Extended-spectrum beta-lactamase (ESBL) and Carbapenemase, sometimes in combination with other resistance mechanisms (e.g., porin loss, efflux pumps, target site alteration) [4,5].

Beta-lactamases, which are produced by bacteria, lead to a variety of beta-lactam antibiotic resistance, including penicillin, cephalosporin, cephamycin, monobactam, and carbapenem [6]. The major *bla*CTX-M, *bla*SHV, *blaTEM*, and minor *blaGES, blaVEB, blaBEL*, and *blaPER* genes that produce ESBL enzymes are capable of hydrolyzing penicillin, monobactams, and third-generation cephalosporins and are inhibited by beta-lactamase inhibitors except for *blaKPC*, which can hydrolyze carbapenem drugs and beta-lactamase inhibitors [7]. The *bla*MOX, *bla*CIT, *bla*DHA, *bla*ACC, *blaEBC,* and *blaFOX* genes, classified as AmpC-beta-lactamases (AmpC-BL), hydrolyze penicillins, monobactams, broad and extended-spectrum cephalosporins, and cephamycin but are sensitive to Cefepime [8,9]. The major *blaNDM-1, blaIMP, blaVIM*, and the minor *blaGIM-1, blaSIM-1, blaAIM-1, blaDIM,* and *blaSPM-1* genes that produce carbapenem-resistant MBL enzymes are metalloproteins and are capable of hydrolyzing all (third-generation cephalosporin, cephamycin, and carbapenem) antibiotics except monobactams [5]. The *blaOXA-23-group, blaOXA-24-group, blaOXA-48,* and *blaOXA-58-group* genes are oxacillinase derivatives, like *blaKPC* and *blaIMI* Carbapenemase, their hydrolytic activity against carbapenems and some 3rd generation cephalosporins, including monobactam, and they are not inhibited by clavulanic acid and tazobactam [5].

Infections with ESBL-producing bacteria have been successfully treated with carbapenem drugs such as Imipenem, Meropenem, Doripenem, and Ertapenem, but the massive use of this antibiotic has accelerated the dissemination of carbapenemase-producing genes [5]. The *Escherichia coli, Klebsiella pneumoniae,* and *Acinetobacter baumannii* are the most common drug-resistant GNB [3]. These strains are frequently overlooked by clinicians without therapeutic options [1]. Although beta-lactam antibiotics are heavily used in many developing countries, the diversity of beta-lactamase and carbapenemase [10] genes is poorly understood [11]. There are a few studies conducted in Ethiopia. Still, the diversity of these drug-resistance genes is not well explored. Therefore, this study aims to further explore the diversity of beta-lactamase and carbapenemase genes in patients admitted to tertiary care hospitals, Tikur Anbessa Specialized Referral Hospital in Addis Ababa, Ethiopia.

## Materials and methods

### Description of study design and research setting

A cross-sectional study was conducted from September 2018 to March 2019 in TASH, Addis Ababa, Ethiopia, which is the largest specialized hospital in the country, with over 700 beds [12]. All patients from all age groups suspected of bloodstream infection, who visited Tikur Anbessa specialized hospital during the study period, who were willing to give blood samples, and who agreed with consent to the study, were included in this study. Convenience sampling techniques were used for all age groups of BSI-suspected patients who volunteered to provide a blood sample and sign a consent form to participate in the study. Patients who refused to participate in the study or had received antibiotics during the previous ten days were excluded.

### Data collection

A standardized questionnaire was used to collect clinical and demographic information, while a trained nurse reviewed the patient’s medical records. The samples were taken by trained laboratory technologists and professional nurses who have previously gathered research data and blood samples. The lead investigator gave all data and sample collectors detailed instructions on how to use pre-made, standardized questionnaires, draw blood, and transport samples to the lab. The principal investigator and expert microbiologists monitored the microbial growth in the blood culture bottle and the rest downstream processes.

### Sample collection, bacterial isolation, and identification

A total of 1486 patient samples were screened, and 231 gram-negative isolates were analyzed. Sample collection, culture, and bacterial identification were initially performed, after which antimicrobial susceptibility testing was carried out. This study was followed by our previous study on phenotypic detection of extended-spectrum beta-lactamase and carbapenem resistance, where the detailed methods are described [13].

### Phenotypic ESBL and AmpC detection

Drug-resistant isolates were initially screened by routine AST. Resistance or intermediate results to ceftazidime, ceftriaxone, or cefotaxime suggested ESBL, and additional resistance to cephamycin (cefotetan) indicated possible AmpC according to CLSI criteria. Breakpoints used for ESBL screening were CAZ ≤ 22 mm, CRO ≤ 25 mm, and CTX ≤ 27 mm. AmpC screening used the same cephalosporins plus cefepime and cefoxitin, with indicative values FEP ≥ 25 mm, and FOX ≤ 18 mm. All cefoxitin-resistant isolates underwent confirmatory testing. ESBL and AmpC were confirmed by E-test following CLSI and EUCAST guidelines [8,14]. E-Test: MIC test strips of ceftazidime ± clavulanic acid and cefotaxime ± clavulanic acid were used. ESBL was confirmed when CTX ≥ 0.5 with CTX/CTL ratio ≥8, or CAZ ≥ 1 with CAZ/CAL ratio ≥8. AmpC testing used cefotetan ± cloxacillin strips; CN/CNI ratio ≥8 indicated AmpC production [15].

### Carbapenem-resistant detection

The modified carbapenemase inactivation method (mCIM) and EDTA-modified mCIM (eCIM) were used to test carbapenem-resistant or intermediate Enterobacteriaceae (Imipenem, Meropenem, Doripenem, Ertapenem) and non-fermenters (Imipenem, Meropenem, Doripenem), excluding Acinetobacter spp. In mCIM, a zone of inhibition of 6–15 mm is positive, 16–18 mm is considered positive with colonies, and ≥19 mm is negative, indicating resistance via non-carbapenemase mechanisms. In eCIM, an increase of ≥5 mm in zone diameter combined with mCIM positivity indicates metallo-beta-lactamase production [14,16].

### DNA extraction for gene detection

Genomic DNA was extracted using a modified boiling method. A 1000 µL aliquot of cell suspension (10⁷ cells/mL) from each GNB was incubated overnight at 37 °C, then centrifuged (4500 rpm, 5 min, 4 °C). Pellets were resuspended in 50 µL nuclease-free water, boiled at 100 °C for 5 min, and centrifuged (3000 g, 10 min). The supernatant was transferred to a new tube, mixed with 0.7 volumes of cold absolute ethanol, and centrifuged (20 min) to precipitate DNA. The pellet was washed with 70% cold ethanol, air-dried, and re-suspended in TE buffer (10 mM Tris-HCl, 1 mM EDTA, pH 8.0) [17]. The final concentration of 70–100ng/μl‌ of the template was prepared by using a Nanodrop Spectrophotometer.

### Conventional PCR for gene detection

The PCR was performed using hot start ready-made Multiplex PCR (5x HOT FIREPol Multiplex Master Mix) containing HOT FIREPol DNA polymerase, 5x multiplex buffer, 10 mM MgCl_2_, dNTPs, and BSA compounds that increase sample density for direct loading were imported from Solis Biodyne company, Estonia, according to the manufacturer’s instructions [18]. All primers were obtained from IDT except AmpC-BL and blaVIM and blaIMP primers, which were imported from LIGO Macrogen Europe. Oligonucleotide yield or lyophilized primer was reconstituted by nuclease-free TE buffer (10 mM Tris, pH 8.0; 0.1 mM EDTA, pH 8.0) using the integrated DNA technology (IDT) protocol (Tables 1 and 2). The PCR amplification was done by using the T3000 Biometra thermocycler, which is the product of Analytik Jena, a company based in Germany.

**Table 1:** Primers used for ESBL and AmpC-BL gene detection.

PCR name	Targeted gene	Primer sequence (5′ to 3′)		Amplicon size in bp	References
**ESBL Multiplex 1 Main**	*blaCTX-M*	Forward	ATGTGCAGYACCAGTAARGTKATGGC	590	[19,20]
		Reverse	GGTRAARTARGTSACCAGAAYCAGCGG		
	*blaTEM*	Forward	TCGCCGCATACACTATTCTCAGAATGAC	422	
		Reverse	CAGCAATAAACCAGCCAGCCGGAAG		
	*blaSHV*	Forward	TGTATTATCTC(C/T)CTGTTAGCC(A/G)CCCTG	739	
		Reverse	GCTCTGCTTTGTTATTCGGGCCAAGC		
**ESBL Multiplex 2 MIN**	*blaPER*	Forward	AGTGTGGGGGCCTGACGAT	725	[19,20]
		Reverse	GCAACCTGCGCAATRATAGCTT		
	*blaGES*	Forward	CTGGCAGGGATCGCTCACTC	600	
		Reverse	TTCCGATCAGCCACCTCTCA		
	*blaBEL*	Forward	CGACAATGCCGCAGCTAACC	448	
		Reverse	CAGAAGCAATTAATAACGCCC		
	*blaVEB*	Forward	CGACTTCCATTTCCCGATGC	376	
		Reverse	TGTTGGGGTTGCCCAATTTT		
**ESBL Multiplex 3 AmpC**	*blaMOXM*	Forward	GCT GCT CAA GGA GCA CAG GAT	520	[21]
		Reverse	CAC ATT GAC ATA GGT GTG GTG C		
	*blaCITM*	Forward	TGG CCA GAA CTG ACA GGC AAA	462	
		Reverse	TTT CTC CTG AAC GTG GCT GGC		
	*blaDHAM*	Forward	AAC TTT CAC AGG TGT GCT GGG T	405	
		Reverse	CCG TAC GCA TAC TGG CTT TGC		
	*blaACCM*	Forward	AAC AGC CTC AGC AGC CGG TTA	346	
		Reverse	TTC GCC GCA ATC ATC CCT AGC		
	*blaEBCM*	Forward	TCG GTA AAG CCG ATG TTG CGG	302	
		Reverse	CTT CCA CTG CGG CTG CCA GTT		
	*blaFOXM*	Forward	AAC ATG GGG TAT CAG GGA GAT G	190	
		Reverse	CAA AGC GCG TAA CCG GAT TGG		

NB: R = A or G; Y = C or T; K = G or T; PCR, polymerase chain reaction; bp, base-pair; ESBL, extended-spectrum beta-lactamase; AmpC, AmpC beta-lactamase.

**Table 2:** Primers used for Carbapenemase and MBL gene detection.

PCR name	Targeted gene	Primer sequence (5′ to 3′)		Amplicon size in bp	References
**Carbapenemase Multiplex 1**	*blaNDM*	Forward	ACTTGGCCTTGCTGTCCTT	603	[20]
		Reverse	CATTAGCCGCTGCATTGAT		
	*blaVIM*	Forward	TGTCCGTGATGGTGATGAGT	437	
		Reverse	ATTCAGCCAGATCGGCATC		
	*blaIMP*	Forward	ACAYGGYTTRGTDGTKCTTG	387	
		Reverse	GGTTTAAYAAARCAACCACC		
	*blaKPC*	Forward	TCGCCGTCTAGTTCTGCTGTCTTG	353	
		Reverse	ACAGCTCCGCCACCGTCAT		
	*blaOXA-48*	Forward	ATGCGTGTATTAGCCTTATCG	265	
		Reverse	CATCCTTAACCACGCCCAAATC		
**Carbapenemase Multiplex 2 OXA**	*blaOXA-23 group*	Forward	CCCCGAGTCAGATTGTTCAAGG	330	
		Reverse	TACGTCGCGCAAGTTCCTGA		
	*blaOXA-24/143 group*	Forward	GCAGAAAGAAGTAAARCGGGT	271	
		Reverse	CCAACCWGTCAACCAACCTA		
	*blaOXA-58 group*	Forward	GGGGCTTGTGCTGAGCATAGT	688	
		Reverse	CCACTTGCCCATCTGCCTTT		
**Carbapenemase Multiplex 3 Mini**	*blaAIM*	Forward	CTGAAGGTGTACGGAAACAC	322	[22]
		Reverse	GTTCGGCCACCTCGAATTG		
	*blaGIM*	Forward	TCGACACACCTTGGTCTGAA	477	
		Reverse	AACTTCCAACTTTGCCATGC		
	*blaSIM*	Forward	TACAAGGGATTCGGCATCG	570	
		Reverse	TAATGGCCTGTTCCCATGTG		
	*blaDIM*	Forward	GCTTGTCTTCGCTTGCTAACG	699	
		Reverse	CGTTCGGCTGGATTGATTTG		
**Carbapenemase-1 additional**	*blaVIM*	Forward	TTTGGTCGCATATCGCAACG	500	
		Reverse	CCATTCAGCCAGATCGGCAT		
	*blaIMP*	Forward	GTTTATGTTCATACWTCG	432	
		Reverse	GGTTTAAYAAAACAACCAC		

D = A, G or T; R = A or G; Y = C or T; K =G or T; W = A or T; PCR, polymerase chain reaction; bp, base-pair; MBL, Metallo-beta-lactamase

### Extended-spectrum beta-lactamase gene detection

The isolates that were screened positive for ESBL were subjected to ESBL multiplex 1 and 2 PCR tests using specific primers (Table 1). In multiplex 1, ESBL, *blaSHV, blaCTX-M,* and *blaTEM* genes were amplified adopting Trung et al.‘s protocol [19]. Multiplex PCR reactions were performed in a final volume of 20 μl containing 4 μl Multiplex master mix, 0.6 μl reverse and 0.6 μl forward primers each containing (10 ng/μl and 0.2 μl), 13.8 μl nuclease-free water, and 1 μl of 70–100 ng/μl DNA template. The thermal cycling conditions were: initial denaturation at 95°C for 240 seconds, followed by 35 cycles of denaturation at 94°C for 25 s, annealing at 58°C for 45 s, extension at 72°C for 1 minute, and final extension at 72°C for 5 min. The multiplex 2 PCR was performed for ESBL, *blaPER, blaGES, blaBEL*, and *blaVEB*, using the protocols of Bogaerts *et al*. [20] (Table 1). The reactions were performed in a final volume of 20 μl containing 4 *μ*l Multiplex master mix, 0.8 μl reverse and 0.8 μl forward primers each containing (10 ng/μl), 13.4 nucleases-free water, and 1 μl of 70–100 ng/μl DNA template. The thermal cycling conditions were: initial denaturation at 95°C for 300 seconds, followed by 30 cycles of denaturation at 94°C for 30 seconds, annealing at 57°C for 90 seconds, extension at 72°C for 90 seconds, and final extension at 72°C for 5 minutes.

### AmpC Beta-Lactamase genes detection

The isolates that were screened positive for AmpC-BLs were subjected to ESBL multiplex 1–2 AmpC-BLs multiplex 3 PCR tests (Table 1). A multiplex 3 PCR was set for AmpC-BLs family *blaMOXM, blaCITM, blaDHAM, blaACCM, blaEBCM,* and *blaFOXM* [21] (Table 1) in a final volume of 20 μl containing 4 μl Multiplex master mix, 1.0 μl reverse, and 1.0 μl forward primers each containing (0.6μM for *blaMOXM, blaCITM, blaDHAM*; 0.5 μM for *blaACCM*, *blaEBCM*, and 0.4 μM for *blaFOXM*), 13.0 μl nuclease-free water, and 1 μl of 70–100 ng/μl DNA template. The thermal cycling conditions were: initial denaturation at 95°C for 240 seconds, followed by 25 cycles of denaturation at 94°C for 30 seconds, annealing at 64°C for 60 seconds, extension at 72°C for 1 minute, and final extension at 72°C for 7 minutes.

### Detection of carbapenemase, oxacillinase, and MBL genes

The carbapenem drug-resistant isolates were subjected to ESBL multiplex PCR 1−3 (Table 2) and carbapenemase multiplex PCR 1−3 (Table 2). Multiplex CARBA PCR 1 was employed in the initial screening test for carbapenemase *blaKPC*, *blaOXA-48*, and metallo-beta-lactamase genes of *blaNDM, blaVIM*, *and blaIMP* [20] (Table 2) in a final volume of 20 μl containing 4 μl Multiplex master mix, 0.6 μl reverse and 0.6 μl forward primers each containing (10 ng/μl), 13.8 μl nuclease-free water, and 1 μl of 70−100 ng/μl DNA template. The thermal cycling conditions were: initial denaturation at 95°C for 300 seconds, followed by 35 cycles of denaturation at 94°C for 30 seconds, annealing at 57°C for 90 seconds, extension at 72°C for 90 seconds, and final extension at 72°C for 300 seconds. Multiplex PCR 2 for OXA family Carbapenemases producing genes of the *blaOXA*-23 group, *blaOXA-24/143* group, and *blaOXA-58* group was carried out using a similar procedure as above [20].

### Minor metallo-beta-lactamase genes

The CARBA multiplex 3 was used for the detection of the minor Metallo-beta-lactamase producing genes, *blaAIM, blaGIM, blaSIM,* and *blaDIM* [22] for carbapenem-resistant strains (Table 2). It was performed in a final volume of 20 μl containing 4 *μ*l Multiplex master mix, 0.8 μl reverse and 0.8 μl forward primers each containing (10 ng/μl), 13.4 μl nuclease-free water, and 1 μl of 70–100 ng/μl DNA template. The thermal cycling conditions were: initial denaturation at 94°C for 300 seconds, followed by 36 cycles of denaturation at 94°C for 30 seconds, annealing at 52°C for 40 seconds, extension at 72°C for 50 seconds, and final extension at 72°C for 350 seconds.

### Quality control

The bacterial identification, AST, and phenotyping characterization of beta-lactamase production test quality controlling procedure were mentioned in the previous paper. DNA samples from reference *blaTEM, blaSHV*, and *blaCTX*-M positive strains were utilized as positive controls for ESBL detection. During PCR analysis, the known laboratory reference *blaKPC, blaOXA-48, and blaNDM* genes were utilized as positive controls, with Escherichia coli ATCC® 25922 serving as the negative control. Each primer pair was tested in monoplex PCR before multiplexing. Before multiplexing the ESBL and carbapenemase primers, each primer was evaluated in a monoplex PCR test.

### Agarose gel electrophoresis

The amplified PCR products from all PCR reactions were loaded in a 1.5% agarose gel containing ethidium bromide and run using 120V, 400mA, for 60 minutes using a Bio-Rad gel electrophoresis machine. DNA bands were visualized using a gel doc Imaging System (Bio-Rad) and results were taken through the internet.

### Statistical analysis and interpretation

Data were collected by trained data collectors, and data quality was ensured through the use of standardized data collection formats and materials. Data captured in EPI INFO were cleaned and analyzed by using SPSS version 24 software for further processing. The quantitative data collected using different techniques were analyzed using simple descriptive statistics. The association was also assessed using the χ^2^-test. In SPSS, sensitivity and specificity are calculated by creating a crosstab between test results and true condition variables, then interpreting the column percentages of true positives and true negatives from the output.

### Ethical clearance

The research received approval from the College of Natural and Computational Sciences Institutional Review Board (CNS-IRB) on March 30, 2018, under minute no. IRB/032/2018 at Addis Ababa University. The College of Health Sciences then accepts this approval letter and gives permission to access their facilities for sample collection. The Helsinki [23] declaration’s requirements were met by this research process. We have obtained written assent (12–18) and written approval from the patient’s legal guardians for participants under the age of 18, but we have obtained patient written consent for those above 18. The study’s objectives and methodologies were communicated to the parents or guardians of the participants during the study period. Patients who provided informed consent were chosen and recruited as study subjects, and their results were disclosed to the attending physicians.

### Operational definition

Drug resistance mechanism (DRM); In these studies, DRM represents ESΒL, AmpC BL, MBL, and Carbapenemase enzyme production to resist one or more of these drug categories called extended-spectrum cephalosporin and/or cephamycin, and carbapenem drug-resistant bacteria.

## Results

### Culture results

Among 1486 samples, 417 culture-positive samples were identified, and 224 (53.7%) of these samples were GNB. Of these, GNB 7 (1.68%) had polymicrobial growth, and a total of 231 GNB were identified. However, among 231 GNB, 195 of them had one or more DRM of these, 189 (81.8%) had good DNA extraction quality, and 6 (2.6%) did not; the remaining 42(18.2%) did not have DRM.

### Phenotypic screening of ESBL, AmpC, Carbapenem, and MBL-resistant bacteria

A total of 188 (81.4%) ESBL and 82 (35.5%) AmpC-BLs were identified phenotypically. Phenotyping confirmatory tests done by E-test reduced from the primary screening results of 188 (81.4%) and 82(35.5%) to 122 (52.8%) and 33 (14.3%) for ESBL and AmpC, respectively. The *Klebsiella pneumoniae*, *Acinetobacter spp,* and *Escherichia coli* GNB showed a higher number of extended-spectrum cephalosporin and cephamycin drug resistance. The number of drug-resistant strains to carbapenem and MBL resistance strains was 74 (32.0%). But during the confirmatory test, it reduced to 69 (29.9%) carbapenem and metallo-beta-lactam drug-resistant strains. The highest carbapenem drug resistance was in *Acinetobacter species* [13].

### Molecular characterization of drug-resistance genes in GNB from BSI

Among the 231 GNB isolates, 189 had phenotypically confirmed DRM, and 176 (76.2%) were PCR-confirmed drug-resistance enzyme-producing strains. In total, 415 drug-resistance–encoding genes were identified in this study. The highest prevalence of resistance was observed in ESBL-producing isolates, followed by MBL, Carbapenemase, and AmpC β-lactamase–producing isolates, each accounting for 117 (50.6%), 50 (21.6%), 46 (19.9%), and 22 (9.5%), respectively. The total carbapenem resistance that produces Carbapenemase and Metalo-beta-lactamase accounted for 63 (27.3%) (Table 3).

**Table 3:** Gram-negative bacteria versus PCR-confirmed drug resistance mechanisms.

GNB	Number of strains	PCR confirmed GNB		PCR confirmed DRM				
		Positive	Negative	ESBL	AmpC	Carbapenemase	MBL	Both Carb-apenemase
	N	N(%)	N(%)	N(%)	N(%)	N(%)	N(%)	N(%)
*K. pneumoniae*	75	70(93.3)	5(6.7)	56(74.7)	6(8)	12(16)	13(17.3)	17(22.7)
*Acinetobacter spp*	47	32(68.1)	15(31.9)	8(17)	2(4.3)	20(42.6)	22(46.8)	25(53.2)
*E. coli*	38	24(63.2)	14(36.8)	17(44.7)	8(21.1)	3(7.9)	5(13.2)	6(15.8)
*K. oxytoca*	27	23(85.2)	4(14.8)	21(77.8)	4(14.8)	3(11.1)	1(3.7)	3(11.1)
*E. coli (A_D)*	11	9(81.8)	2(18.2)	7(63.6)	0(0)	1(9.1)	2(18.2)	2(18.2)
*C. diversus*	5	2(40)	3(60)	2(40)	0(0)	0(0)	0(0)	0(0)
*Pseudomonas spp*	5	3(60)	2(40)	0(0)	0(0)	0(0)	2(40)	2(40)
*K. rhinoscleromatis*	4	2(50)	2(50)	0(0)	0(0)	2(50)	1(25)	2(50)
*S. marcescens*	4	3(75)	1(25)	1(25)	1(25)	1(25)	2(50)	2(50)
*K. ozaenae*	3	1(33.3)	2(66.7)	1(33.3)	1(33.3)	0(0)	0(0)	0(0)
*E. cloacae*	2	1(50)	1(50)	0(0)	0(0)	1(50)	1(50)	1(50)
*E. agglomerans*	2	1(50)	1(50)	1(50)	0(0)	0(0)	0(0)	0(0)
*M. morganii*	2	1(50)	1(50)	0(0)	0(0)	1(50)	1(50)	1(50)
*P. mirabilis*	2	1(50)	1(50)	1(50)	0(0)	1(50)	0(0)	1(50)
*P. rettgeri*	2	2(100)	0(0)	2(100)	0(0)	0(0)	0(0)	0(0)
*E. aerogenes*	1	1(100)	0(0)	0(0)	0(0)	1(100)	0(0)	1(100)
*S. typhi*	1	0(0)	1(100)	0(0)	0(0)	0(0)	0(0)	0(0)
**Total**	**231**	**176(76.2)**	**55(23.8)**	**117(50.6)**	**22(9.5)**	**46(19.9)**	**50(21.6)**	**63(27.3)**

NB: GNB, Gram-negative bacteria; DRM, Drug resistance mechanisms; ESBL, extended-spectrum beta-lactamase; AmpC, AmpC beta-lactamase; MBL, Metallo-beta-lactamase.

The number of AST-confirmed DRM was reduced by PCR-confirmed DRM encoding gene from ESBL 132(57.1%) to 123 (53.2%), AmpC 36(15.6%) to 22(9.5%), and Carbapenemase with MBL producer 69(29.9%) was reduced to 63(27.3%) (Table 4).

**Table 4:** Summary of DRM for all drug-resistant enzyme-producing gram-negative bacteria.

Method for screening DRM	DRM among 231 strains					Total Strains
	ESBL	AmpC	Carba	MBL	Both	
**N(%)**	**N(%)**	**N(%)**	**N(%)**	**N(%)**	**N**
Phenotyping detection	122 (52.8)	33 (14.3)	58 (25.1)	13 (5.6)	69 (29.9)	231
PCR detection	117 (50.6)	22 (9.5)	46 (19.9)	50 (21.6)	63 (27.3)	231

NB: GNB, Gram-negative bacteria; DRM, Drug resistance mechanisms; ESBL, extended-spectrum beta-lactamase; AmpC, AmpC beta-lactamase; MBL, Metallo-beta-lactamase.

The chi-square analysis indicates a strong association between phenotypic and genotypic characterization of DRM. For all comparisons between phenotypic and genotypic ESBL, AmpC, carbapenemase, and MBLs DRM p-value was < 0.0001 (Table 5).

**Table 5:** Crosstabulation of Phenotyping versus Genotyping drug-resistant mechanism.

Method for screening DRM		Genotyping resistant		Total	Chi-Square test		
		No N(%)	Yes N(%)	N(%)	Value	df	P-value
Phenotyping ESBL confirmed	No N(%)	67(93.1)	0(0)	67(35.4)	152.098	1	<0.0001
	Yes N(%)	5(6.9)	117(100)	122(64.6)			
Total	N(%)	72(100)	117(100)	189(100)			
Phenotyping AmpC confirmed	No N(%)	156(93.4)	0(0)	156(82.5)	105.817	1	<0.0001
	Yes N(%)	11(6.6)	22(100)	33(17.5)			
Total	N(%)	167(100)	22(100)	189(100)			
Phenotyping Carbapenem-Resistant	No N(%)	126(88.1)	7(15.2)	133(70.4)	88.696	1	<0.0001
	Yes N(%)	17(11.9)	39(84.8)	56(29.6)			
Total	N(%)	143(100)	46(100)	189(100)			
Phenotyping MBLs Resistant	No N(%)	138(99.3)	38(76.0)	176(93.1)	31.116	1	<0.0001
	Yes N(%)	1(0.7)	12(24.0)	13(6.9)			
Total	N(%)	139(100)	50(100)	189(100)			

NB: GNB, Gram-negative bacteria; DRM, Drug resistance mechanisms; ESBL, extended-spectrum beta-lactamase; AmpC, AmpC beta-lactamase; MBL, Metallo-beta-lactamase.

### Molecular identification of ESBL coding genes

**Fig 1:**
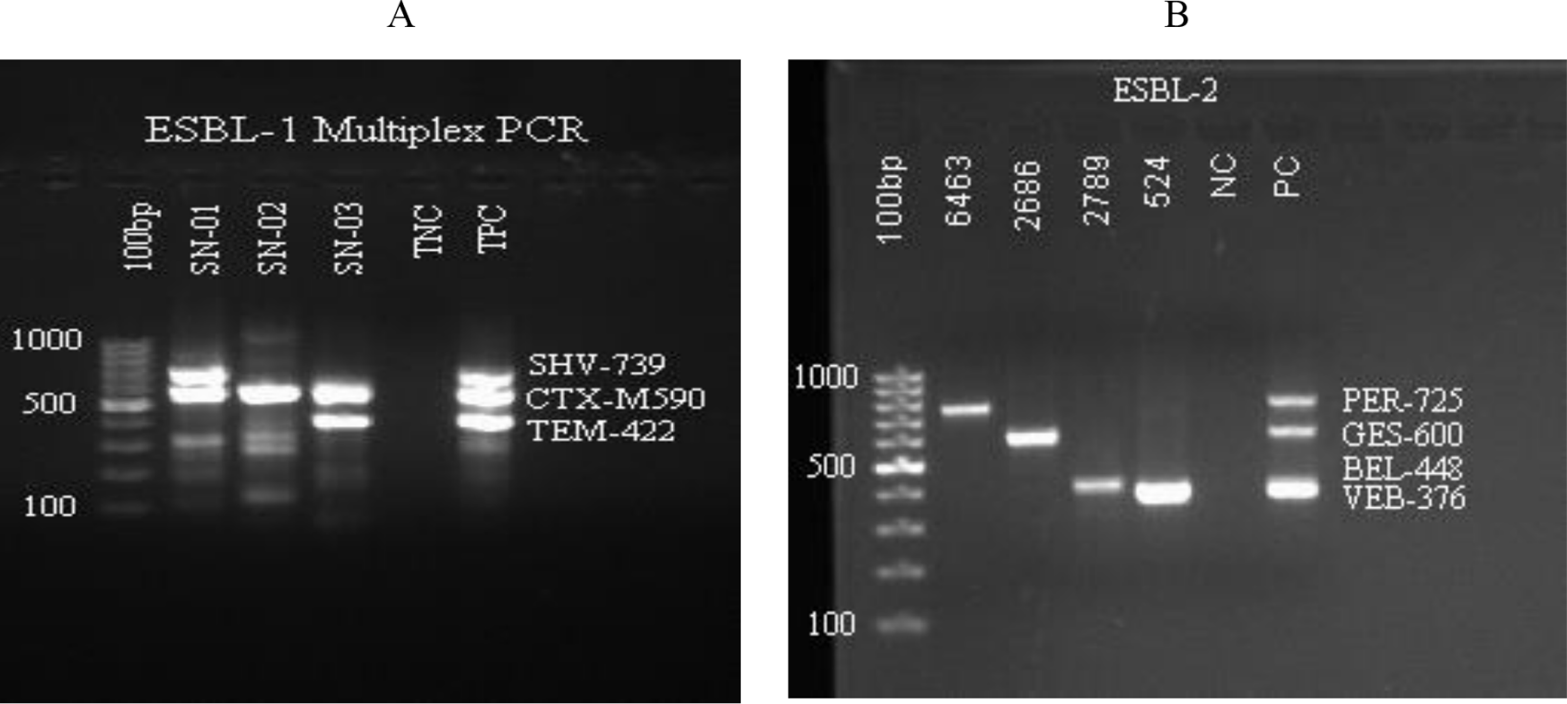
Multiplex PCR ESBL 1 and ESBL 2 Gel doc reading. a). ESBL multiplex PCR 1, Major ESBL for the detection of *blaSHV*, *blaCTXM*, and *blaTEM* genes from 3 patient samples with TNC-negative and TPC-positive control. b). ESBL multiplex PCR 2, Minor ESBL for the detection of *blaPER*, *blaGES*, *blaBEL*, and *blaVEB* genes from 4 patient samples with NC-negative and PC-positive control.

Overall, 52.8% (122/231) of isolates were phenotypically ESBL positive for the E-test, and 95.9% (117/122) of these were confirmed positive by PCR, with 100% sensitivity and 86.4% specificity (Table 6). Major ESBL detection was used to identify *blaSHV, blaCTXM, and blaTEM* genes, whereas minor ESBL was used to detect *blaPER, blaGES, blaBEL*, and *blaVEB* genes (Fig 1). The *blaCTX-M*, detected in 85.2% (104/122) of isolates, was the most prevalent ESBL gene, followed by *blaTEM* 79.5% (97/122) and *blaBEL* 17.2% (21/122). The genes *blaSHV*, *blaGES, blaVEB,* and *blaPER* were detected at lower frequencies in this study. Statistical analysis demonstrated significant associations for *blaTEM* (p < 0.0001), *blaCTX-M* (p < 0.0001), *blaSHV* (p = 0.002), *blaVEB* (p = 0.005), and total PCR ESBL (p = 0.010*), indicating these genes are significantly distributed among the ESBL producers. Genes *blaGES* and *blaBEL* did not show statistically significant differences (p > 0.05) (Table 6).

**Table 6:** Gram-negative bacteria versus PCR-confirmed ESBL drug resistance genes.

Genus	Species	Total GNB	E-test Confirmed ESBL	ESBL gene positive among 231 GNB							PCR Confirmed ESBL	
				*bla TEM 22*	*bla CTX-M 590*	*bla SHV 739*	*bla VEB 376*	*bla BEL 448*	*bla GES 600*	*bla PER 725*	Negative	Positive
			N(%)	N(%)	N(%)	N(%)	N(%)	N(%)	N(%)	N(%)	N(%)	N(%)
*Klebsiella spp*	*K. pneumoniae*	75	58(77.3)	51(87.9)	51(87.9)	8(13.8)	2(3.4)	6(10.3)	4(6.9)	0(0)	2(3.4)	56(96.6)
	*K. oxytoca*	27	21(77.8)	21(100)	21(100)	2(9.5)	0(0)	3(14.3)	2(9.5)	0(0)	0(0)	21(100)
	*K. ozaenae*	3	1(33.3)	1(100)	0(0)	1(100)	0(0)	0(0)	0(0)	0(0)	0(0)	1(100)
	*K. rhinoscleromatus*	4	0(0)	0(0)	0(0)	0(0)	0(0)	0(0)	0(0)	0(0)	0(0)	0(0)
	**Total**	**109**	80(73.4)	73(91.3)	72(90)	11(13.8)	2(2.5)	9(11.3)	6(7.5)	0(0)	2(2.5)	78(97.5)
	*Acinetobacter spp*	47	10(21.3)	0(0)	2(20)	0(0)	2(20)	5(50)	1(10)	0(0)	2(20)	8(80)
*Enterobacter spp*	*E. arogenes*	1	0(0)	0(0)	0(0)	0(0)	0(0)	0(0)	0(0)	0(0)	0(0)	0(0)
	*E. cloacae*	2	0(0)	0(0)	0(0)	0(0)	0(0)	0(0)	0(0)	0(0)	0(0)	0(0)
	*E. agglumerance*	2	1(50)	1(100)	1(100)	0(0)	1(0)	0(0)	0(0)	0(0)	0(0)	1(100)
	**Total**	**5**	1(20)	1(100)	1(100)	0(0)	1(100)	0(0)	0(0)	0(0)	0(0)	1(100)
*Escherichia Spp*	*E. coli*	38	17(44.7)	11(64.7)	16(94.1)	0(0)	1(5.9)	5(29.4)	5(29.4)	1(5.9)	0(0)	17(100)
	*E. coli (A_D)*	11	7(63.6)	7(100)	7(100)	0(0)	1(14.3)	2(28.6)	0(0)	0(0)	0(0)	7(100)
	**Total**	**49**	24(49)	18(75)	23(95.8)	0(0)	2(8.3)	7(29.2)	5(20.8)	1(4.2)	0(0)	24(100)
Other GNB	*C. Diversus*	5	2(40)	2(100)	2(100)	0(0)	0(0)	0(0)	0(0)	0(0)	0(0)	2(100)
	*M. morgani*	2	0(0)	0(0)	0(0)	0(0)	0(0)	0(0)	0(0)	0(0)	0(0)	0(0)
	*P. mirabillis*	2	1(50)	0(0)	1(100)	1(100)	0(0)	0(0)	0(0)	0(0)	0(0)	1(100)
	*P. rettegri*	2	2(100)	2(100)	2(100)	0(0)	1(50)	0(0)	0(0)	0(0)	0(0)	2(100)
	*S. typhi*	1	0(0)	0(0)	0(0)	0(0)	0(0)	0(0)	0(0)	0(0)	0(0)	0(0)
	*S. marcescens*	4	2(50)	1(50)	1(50)	0(0)	0(0)	0(0)	0(0)	0(0)	1(50)	1(50)
	*Pseudomonas spp*	5	0(0)	0(0)	0(0)	0(0)	0(0)	0(0)	0(0)	0(0)	0(0)	0(0)
	**Total**	**21**	7(33.3)	5(71.4)	6(85.7)	1(14.3)	1(14.3)	0(0)	0(0)	0(0)	1(14.3)	6(85.7)
	**Grand Total**	**231**	**122(52.8)**	**97(79.5)**	**104(85.2)**	**12(9.8)**	**8(6.6)**	**21(17.2)**	**12(9.8)**	**1(0.82)**	**5(4.1)**	**117(95.9)**
	Chi-square		26.321	61.882	47.079	31.405	28.248	13.208	11.592			26.321
	df		12	12	12	12	12	12	12			12
	**Sig.**		.010*	.000*	.000*	.002*	.005*	**0.354**	**0.479**			0.010*

**NB:** GNB, Gram-negative bacteria; ESBL, extended-spectrum beta-lactamase

*Klebsiella species* accounted for 47% (109/231) of all gram-negative bacterial isolates. Of these isolates, 73.3% (80/109) were E-test confirmed ESBL producers, while PCR confirmed were 97.5% (78/80). In this study, 77.3% (58/75) of *Klebsiella pneumoniae* isolates were identified as ESBL producers using the E-test, and 96.6% (56/58) of these were confirmed to carry ESBL genes by PCR. Among the *Klebsiella pneumoniae* isolates, the most commonly detected ESBL genes were *blaTEM* and *blaCTX-M*, each found in 51 of 58 isolates (87.9%), followed by *blaSHV*, identified in 8 of 58 isolates (13.8%). Out of 27 *Klebsiella oxytoca* isolates, 21 were confirmed as ESBL producers by E-test, and all of which tested positive by PCR. Both *blaTEM* and *blaCTX-M* genes were detected in all PCR-positive isolates. Other *Klebsiella species,* such as *Klebsiella ozaenae* and *Klebsiella rhinoscleromatis,* were less frequent (Table 6).

A total of 49 *Escherichia spp* isolates were found, of which 24 (49%) were phenotypically confirmed as ESBL producers using the E-test, and all were confirmed positive by PCR. The most frequently detected gene was *blaCTX-M* 95.8% (23/24), followed by *blaTEM* 75% (18/24) (P < 0.0001) and *blaBEL* 29.2% (7/24). In this study, 47 *Acinetobacter spp*. Isolates were identified, of which 21.3% (10/47) were confirmed as ESBL producers by E-test. Among these, 80% (8/10) were PCR-positive, with *blaBEL* being the most commonly detected gene, identified in 50% (5/10) of the ESBL-producing isolates. Other gram-negative bacteria made up 9% (n = 21) of isolates, with 33.3% (7/21) ESBL producers phenotypically and 85.7% (6/7) PCR-positive for ESBL genes (Table 6).

### Molecular identification of AmpC-BLs encoding genes

**Fig 2:**
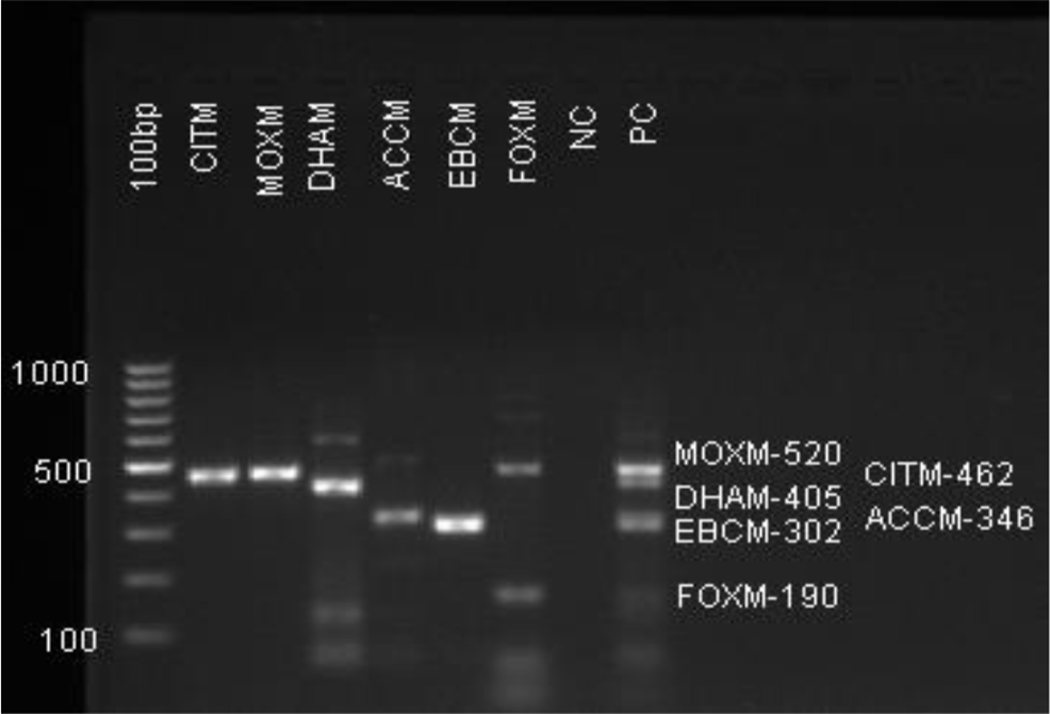
Multiplex PCR ESBL and AmpC 3 Gel doc reading. ESBL Multiplex PCR 3, AmpC-BL gene detection of *bla*MOXM, *bla*CITM, *bla*DHAM, *bla*ACCM, *blaEBCM,* and *blaFOXM* genes of 6 patient samples with NC-negative and PC-positive control.

Among the 231 Gram-negative bacterial isolates, 14.3% (33/231) were phenotypically identified as AmpC producers. Of these, 66.7% (22/33) were confirmed positive for AmpC genes by PCR. The presence of AmpC β-lactamase genes predicted phenotypic resistance with 100% sensitivity and 92% specificity. AmpC-BL gene detection was done for the detection of *bla*MOXM, *bla*CITM, *bla*DHAM, *bla*ACCM, *blaEBCM,* and *blaFOXM* genes (Fig 2). The most frequently detected gene was *blaFOXM*, found in 39.4% (13/33) of the isolates, followed by *blaCITM* in 36.4% (12/33). *blaMOXM* and *blaEBCM* were each detected in 15.2% (5/33) of the isolates, with *blaEBCM* showing a statistically significant association (p = 0.02), and overall PCR AmpC positivity (p = 0.045), suggesting these findings are unlikely due to chance (Table 7).

**Table 7:** Gram-negative bacteria versus PCR-confirmed AmpC drug resistance genes.

Genus	Species	Total GNB	E-test confirmed Ampc	AmpC gene positive among 231 GNB						PCR confirmed AmpC
				*blaMOXM 520*	*blaCITM 436*	*blaDHAM 405*	*blaACCM 346*	*blaEBCM 302*	*blaFOXM 190*	
		N	N (%)	N (%)	N (%)	N (%)	N (%)	N (%)	N (%)	N&%
*Klebsilla species*	*K. pneumoniae*	75	6(8)	1 (16.7)	4 (66.7)	0 (0)	0 (0)	2 (33.3)	5 (83.3)	6(100)
	*K. oxytoca*	27	4(14.8)	1 (25)	0 (0)	0 (0)	2 (50)	0 (0)	1 (25)	4(100)
	*K. ozaenae*	3	1(33.3)	1 (100)	0 (0)	0 (0)	0 (0)	1 (100)	0 (0)	1(100)
	*K. rhinoscleromatus*	4	0(0)	0 (0)	0 (0)	0 (0)	0 (0)	0 (0)	0 (0)	0(0)
	**Total**	**109**	11(10.1)	3 (27.3)	4 (36.4)	0 (0)	2 (18.2)	3 (27.3)	6 (54.5)	11(100)
	Acinetobacter spp	47	9(19.1)	1 (11.1)	1 (11.1)	0 (0)	0 (0)	0 (0)	1 (11.1)	2(22.2)
*Enterobacter species*	*E. arogenes*	1	1(100)	0 (0)	0 (0)	0 (0)	0 (0)	0 (0)	0 (0)	0(0)
	*E. cloacae*	2	0(0)	0 (0)	0 (0)	0 (0)	0 (0)	0 (0)	0 (0)	0(0)
	*E. agglumerance*	2	0(0)	0 (0)	0 (0)	0 (0)	0 (0)	0 (0)	0 (0)	0(0)
	**Total**	**5**	1(20)	0 (0)	0 (0)	0 (0)	0 (0)	0 (0)	0 (0)	0(0)
*Escherichia Coli.*	*E. coli*	38	8(21.1)	1 (12.5)	7 (87.5)	1 (12.5)	1 (12.5)	1 (12.5)	6 (75)	8(100)
	*E. coli (A_D)*	11	1(9.1)	0 (0)	0 (0)	0 (0)	0 (0)	0 (0)	0 (0)	0(0)
	**Total**	**49**	9(18.4)	1 (11.1)	7 (77.8)	1 (11.1)	1 (11.1)	1 (11.1)	6 (66.7)	8(88.9)
Other GNB	*C. Diversus*	5	0(0)	0 (0)	0 (0)	0 (0)	0 (0)	0 (0)	0 (0)	0(0)
	*M. morgani*	2	0(0)	0 (0)	0 (0)	0 (0)	0 (0)	0 (0)	0 (0)	0(0)
	*P. mirabillis*	2	0(0)	0 (0)	0 (0)	0 (0)	0 (0)	0 (0)	0 (0)	0(0)
	*P. rettegri*	2	0(0)	0 (0)	0 (0)	0 (0)	0 (0)	0 (0)	0 (0)	0(0)
	*S. typhi*	1	0(0)	0 (0)	0 (0)	0 (0)	0 (0)	0 (0)	0 (0)	0(0)
	*S. marcescens*	4	1(25.0)	0 (0)	0 (0)	0 (0)	0 (0)	1 (100)	0 (0)	1(25)
	*Pseudomonas spp*	5	2(40)	0 (0)	0 (0)	0 (0)	0 (0)	0 (0)	0 (0)	0(0)
	**Total**	**21**	3(14.3)	0 (0)	0 (0)	0 (0)	0 (0)	1 (33.3)	0 (0)	1 (33.3)
	**Grand Total**	**231**	**33(14.3)**	**5 (15.2)**	**12 (36.4)**	**1 (3)**	**3 (9)**	**5 (15.2)**	**13 (39.4)**	22(66.7)
	Chi-square			7.363	11.813	2.674	11.127	15.097	7.718	12.857
	df			6	6	6	6	6	6	6
	Sig.			0.289	0.066	0.848	0.085	0.02	0.26	0.045

**NB:** PCR, polymerase chain reaction; AmpC, AmpC-beta-lactamase; GNB, gram-negative bacteria.

Among 109 *Klebsiella isolates*, 10.1% were phenotypically positive for AmpC β-lactamase production, all of which were confirmed by PCR. The most frequently detected gene was *blaFOX-M*, present in 54.5% (6/11) of the phenotypically positive isolates, followed by *blaCIT-M* in 36.4% (4/11), and *blaEBC-M* and *blaMOXM* in 27.3% (3/11) (Table 7).

A total of 49 *Escherichia coli* isolates were identified in this study, of which 18.4% (9/49) were phenotypically confirmed as AmpC producers. Among these, 88.9% (8/9) were PCR-positive for AmpC genes. The most frequently detected gene in *Escherichia coli* was *blaCITM*, found in 77.8% (7/9) of the isolates, followed by *blaFOXM* at 66.7% (6/9). Other genes, including ***b****laMOXM*, ***b****laDHAM*, *blaACCM*, and *blaEBCM*, were detected at lower frequencies. Additionally, 47 *Acinetobacter species* isolates were identified, with 19.1% (9/47) phenotypically AmpC producers. PCR confirmed AmpC genes in 22.5% of these isolates. The genes *blaFOXM*, *blaMOXM*, and *blaCITM* were each detected in 11.1% of the PCR-positive *Acinetobacter isolates* (Table 7).

### Molecular identification of the carbapenemase-encoding gene

**Fig 3:**
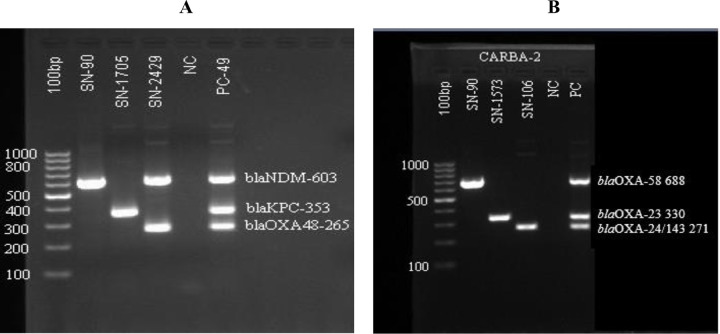
Multiplex PCR CARBA 1 and Multiplex PCR CARBA 2 Gel doc reading. a). CARBA Multiplex PCR 1, Carbapenemase gene detection of *bla*NDM, *blaKPC,* and *blaOXA-48*, genes of 3 patient samples with NC-negative and PC-positive control. b). CARBA Multiplex PCR 2, Oxacillinase gene detection of *bla*OXA-58, *blaOXA-23,* and *blaOXA24/143*, genes of 3 patient samples with NC-negative and PC-positive control.

**Fig 4:**
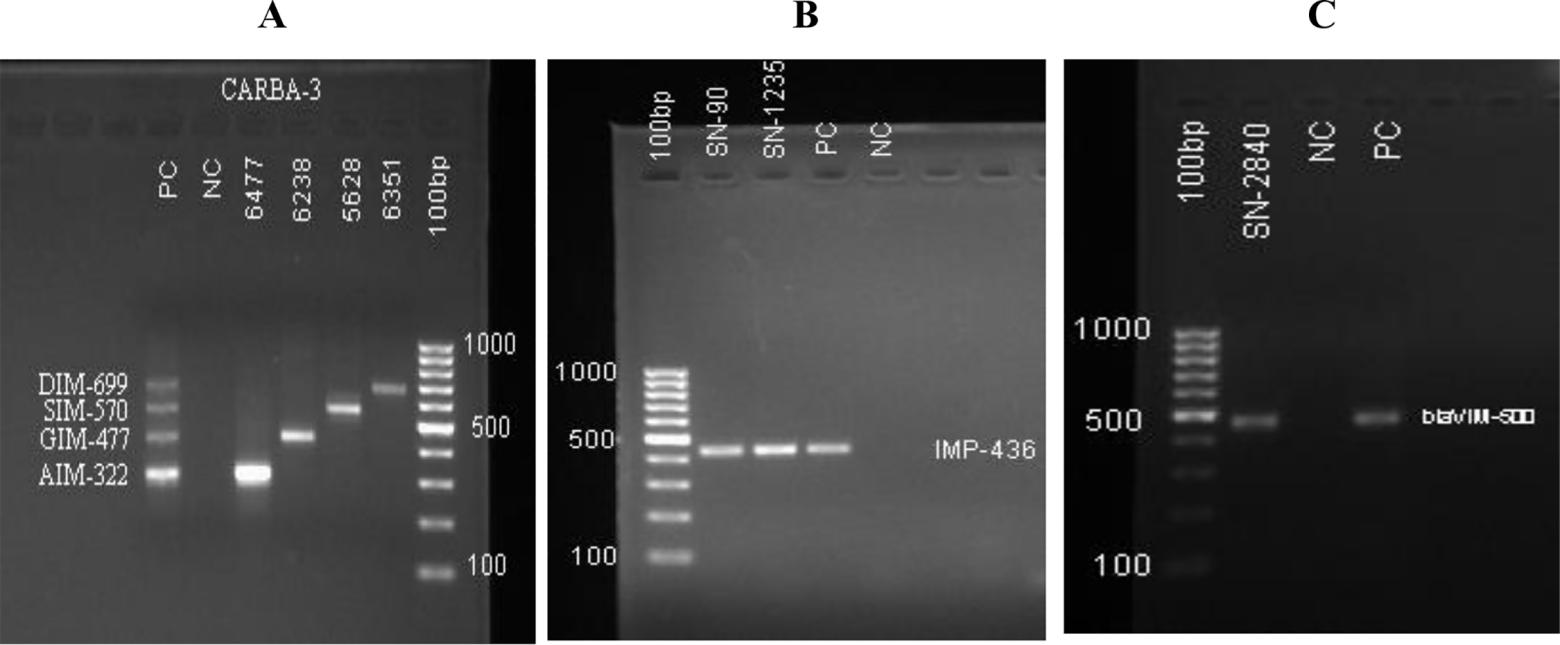
Multiplex PCR CARBA 3 Gel doc reading. **a)**.CARBA Multiplex PCR 3, Minor MBL gene detection of *bla*DIM, *bla*SIM, *bla*GIM, and *blaAIM* genes of 4 patient samples with NC-negative and PC-positive control. **b)**. CARBA Multiplex PCR 1, MBL gene detection of *blaIMP* genes of patient samples with NC-negative and PC-positive control. **c)**. CARBA Multiplex PCR 1, MBL gene detection of *blaVIM* genes of patient samples with NC-negative and PC-positive control.

A total of 69 (29.9%) Gram-negative bacilli (GNB) were identified as carbapenemase producers using the modified carbapenem inactivation method (mCIM) and the EDTA-modified carbapenem inactivation method (eCIM). Of these, 63 isolates (91.3%) were confirmed by PCR to harbor carbapenemase genes. Among the PCR-positive isolates, 46 (66.6%) carried carbapenemase-encoding genes, and 50 (72.5%) possessed metallo-β-lactamase genes. PCR exhibited a specificity of 88% and a sensitivity of 85% in predicting phenotypic carbapenem resistance. Carbapenemase gene detection was done for *blaKPC*, and Oxacillinase gene detection was done for *blaOXA-48, blaOXA-58, blaOXA-23,* and *blaOXA24/143*. Major MBL gene detection was done for *blaNDM,* (Fig 3) *blaIMP,* and *blaVIM,* whereas Minor MBL gene detection was done for *blaDIM, blaSIM, blaGIM,* and *blaAIM* genes (Fig 4).

The most prevalent carbapenemase genes in this study were *blaOXA-23*, detected in 40.6% (28/69) of isolates, followed by *blaNDM* in 39.1% (27/69), and *blaVIM* in 34.8% (26/69). Other genes, such as *blaKPC* and both *blaOXA-58* and *blaOXA-48*, were present in approximately 14.5% and 10.1% of isolates, respectively. Other genes like *blaIMP* and *blaAIM* (each 7.2%), *blaGIM* (5.8%), *blaSIM* (4.3%), and *blaOXA-24/143* (1.4%) were less frequently detected. Statistical analysis showed significant associations for c *blaOXA-48* (p = 0.008) and *blaNDM* (p = 0.002), indicating these genes’ distribution among isolates is unlikely due to chance (Table 8).

**Table 8:** Gram-negative bacteria versus PCR-confirmed Carbapenem drug resistance genes.

*Genus*	Species	Total GNB	MIC conf. Carba	PCR-results													GNB Carba & MBL positive
				Carbapenemase producer genes					GNB Carba positive	Metallo-β-lactamase genes						GNB MBL positive	
				*blaKPC 353*	*blaOXA48 265*	*blaOXA −23 330*	*blaOXA-24/143 271*	*blaOXA-58 688*		*blaNDM 603*	*blaVIM 437*	*blaIMP 387*	*blaAIM 322*	*blaGIM 477*	*blaSIM 570*		
**N (%)**	**N (%)**	**N (%)**	**N (%)**	**N (%)**	**N (%)**	**N (%)**	**N (%)**	**N (%)**	**N (%)**	**N (%)**	**N (%)**	**N (%)**	**N (%)**
*Klebsiella species*	*K. pneumoniae*	75	18 (24)	6 (33)	1 (6)	5 (28)	0 (0)	0 (0)	12 (66.7)	13 (72)	0 (0)	3 (16.7)	0 (0)	1 (5.6)	1 (5.6)	13(72.2)	17(94.4)
	*K. oxytoca*	27	3 (11)	2 (66.7)	1 (33.3)	1 (33.3)	0 (0)	0 (0)	3 (100)	0 (0)	1 (33.3)	0 (0)	0 (0)	0 (0)	1 (33.3)	1(33.3)	3(100)
	*K. ozaenae*	3	0 (0)	0 (0)	0 (0)	0 (0)	0 (0)	0 (0)	0 (0)	0 (0)	0 (0)	0 (0)	0 (0)	0 (0)	0 (0)	0(0)	0(0)
	*K. rhinoscleromatis*	4	2 (50)	0 (0)	0 (0)	1 (50)	0 (0)	1 (50)	2 (100)	1 (50)	0 (0)	0 (0)	0 (0)	0 (0)	0 (0)	1(50)	2(100)
	**Total**	**109**	23 (21)	8 (34.8)	2 (8.7)	7 (30.4)	0 (0)	1 (4.3)	17 (73.9)	14 (60)	1 (4.3)	3 (13)	0 (0)	1 (4.3)	2 (8.7)	15(65.2)	22(95.6)
	*Acinetobacter spp*	**47**	29 (61.7)	1 (3.4)	2 (6.9)	16 (55.2)	1 (3.4)	4 (13.8)	20 (68.9)	8 (27.6)	18 (62.1)	0 (0)	1 (3.4)	2 (6.9)	1 (3.4)	22(75.9)	25(86.2)
*Enterobacter species*	*E. aerogenes*	1	1 (100)	0 (0)	1 (100)	0 (0)	0 (0)	0 (0)	1 (100)	0 (0)	0 (0)	0 (0)	0 (0)	0 (0)	0 (0)	0(0)	1(100)
	*E. cloacae*	2	1 (50)	0 (0)	0 (0)	1 (100)	0 (0)	0 (0)	1 (100)	0 (0)	1 (100)	0 (0)	0 (0)	0 (0)	0 (0)	1(100)	1(100)
	*E. agglomerans*	2	0 (0)	0 (0)	0 (0)	0 (0)	0 (0)	0 (0)	0 (0)	0 (0)	0 (0)	0 (0)	0 (0)	0 (0)	0 (0)	0(0)	0(0)
	**Total**	**5**	2 (40)	0 (0)	1 (50)	1 (50)	0 (0)	0 (0)	2 (100)	0 (0)	1 (50)	0 (0)	0 (0)	0 (0)	0 (0)	1(50)	2(100)
*Escherichia Coli.*	*E. coli*	38	6 (15.7))	1 (16.7)	0 (0)	1 (16.7)	0 (0)	1 (16.7)	3 (50)	3 (50)	2 (33.3)	1 (16.7)	2 (33.3)	0 (0)	0 (0)	5(83.3)	6(100)
	*E. coli (A_D)*	11	2 (18)	0 (0)	1 (50)	1 (50)	0 (0)	0 (0)	1 (50)	1 (50)	0 (0)	0 (0)	1 (50)	1 (50)	0 (0)	2(100)	2(100)
	**Total**	**49**	8 (16)	1 (12.5)	1 (12.5)	2 (25)	0 (0)	1 (12.5)	4 (50)	4 (50)	2 (25)	1 (12.5)	3 (38.5)	1 (12.5)	0 (0)	7(87.5)	8(100)
Other GNB	*C. Diversus*	5	0 (0)	0 (0)	0 (0)	0 (0)	0 (0)	0 (0)	0 (0)	0 (0)	0 (0)	0 (0)	0 (0)	0 (0)	0 (0)	0(0)	0(0)
	*M. morganii*	2	1 (50)	0 (0)	0 (0)	0 (0)	0 (0)	1 (100)	1 (100)	1 (100)	0 (0)	0 (0)	0 (0)	0 (0)	0 (0)	1(100)	1(100)
	*P. mirabillis*	2	1(50)	0 (0)	1 (100)	1 (100)	0 (0)	0 (0)	1 (100)	0 (0)	0 (0)	0 (0)	0 (0)	0 (0)	0 (0)	0(0)	1(100)
	*P. rettegri*	2	0 (0)	0 (0)	0 (0)	0 (0)	0 (0)	0 (0)	0 (0)	0 (0)	0 (0)	0 (0)	0 (0)	0 (0)	0 (0)	0(0)	0(0)
	*S. typhi*	1	0 (0)	0 (0)	0 (0)	0 (0)	0 (0)	0 (0)	0 (0)	0 (0)	0 (0)	0 (0)	0 (0)	0 (0)	0 (0)	0(0)	0(0)
	*S. marcescens*	4	2 (50)	0 (0)	0 (0)	1 (50)	0 (0)	0 (0)	1 (50)	0 (0)	2 (100)	0 (0)	0 (0)	0 (0)	0 (0)	2(100)	2(100)
	*Pseudomonas spp*	5	3 (60)	0 (0)	0 (0)	0 (0)	0 (0)	0 (0)	0 (0)	0 (0)	0 (0)	1 (33.3)	1(33.3)	0 (0)	0 (0)	2(66.7)	2(66.7)
	**Total**	21	7 (33.3)	0 (0)	1 (14.3)	2 (28.6)	0 (0)	1 (14.3)	3 (42.8)	1 (14.3)	4 (57.1)	1 (14.3)	1 (14.3)	0 (0)	0 (0)	5(71.4)	6(85.7)
	**Grand Total**	**231**	**69 (29.8)**	**10(14.5)**	**7(10.1)**	**28(40.6)**	**1 (1.4)**	**7(10.1)**	**46(66.7)**	**27(39.1)**	**24 (34.8)**	**5(7.2)**	**5 (7.2)**	**4 (5.8)**	**3 (4.3)**	**50(72.5)**	**63(91.3)**
	X^2^			16.827	25.414	11.837	1.4	16.546	11.819	19.021	30.085	9.488	17.44	8.453	7.044	11.72	
	df			11	11	11	11	11	11	11	11	11	11	11	11	11	
	**Sig.**			**.113a**	**.008**	**.376**	**1.0**	**.122**	**.377**	**.061**	**.002**	**.57**	**.096**	**.672**	**.796**	**.364**	

NB: PCR, polymerase chain reaction; Carba, Carbapenemase; MBL, Metallo-beta lactamase; GNB, Gram-negative bacteria; mCIM, modified carbapenemase inactivation method; eCIM, EDTA-modified carbapenemase inactivation method; conf., confirmed.

*Klebsiella pneumoniae* (n = 75) showed 24% (18/75) carbapenemase positivity by CIM, and 94.4% (17/18) of these were positive by PCR, and metallo-beta-lactamase producing genes were 66.7% (12/18) and 72.2%(13/18), respectively. Among *Klebsiella pneumoniae* isolates, the most prevalent carbapenemase gene was *blaKPC*, detected in 33% (6/18) of isolates, followed by *blaOXA-23* in 28% (5/18), and *blaIMP* in 16.7% (3/18).

Among Acinetobacter spp. (n = 47), 61.7%(29/47) were tested positive for capbapenemase production by CIM. Of these CIM-positive isolates, 86.2% (25/29) were confirmed by PCR to carry carbapenemase and metallo-β-lactamase encoding genes. Of these, 68.9% (20/29) isolates carried carbapenemase, and 75.9% (22/29) isolates carried metallo-beta-lactamase-producing genes. The most frequently detected genes were *blaVIM,* present in 62.1% (18/29) of the *Acinetobacter spp* isolates, followed by *blaOXA-23,* 55.2% (16/29), and *blaNDM,* 27.6% (8/29) (Table 6).

Among *Escherichia coli* isolates (n = 38), 15.7% (6/38) tested positive for carbapenemase production using the CIM test, all of which were confirmed by PCR. Of these PCR-positive isolates, 50% (3/6) harbored carbapenemase-encoding genes, and 83.3% (5/6) carried metallo-β-lactamase genes. The most frequently detected gene was *blaNDM*, found in 50% (3/6) of the isolates, followed by *blaVIM* and *blaAIM*, each detected in 33.3% (2/6) (Table 8).

Other Gram-negative bacteria (n = 21) had 33.3% (7/21) AST positivity, with lower gene detection rates. Overall, *blaOXA*-*23* 40.6% (28/69), *blaNDM* 39.1% (27/69), and *blaVIM* 34.8% (24/69) were the most prevalent carbapenemase genes detected by PCR. In contrast, other carbapenemase genes, such as *blaKPC*, *blaOXA*-23, *blaNDM*, *blaIMP*, *blaAIM*, *blaGIM*, and *blaSIM*, despite some having moderate prevalence, did not show statistically significant associations (p > 0.05), suggesting a more random or less impactful distribution within this sample (Table 8).

## Discussion

Antibiotic-resistant Enterobacteriaceae have a significant impact on clinical outcomes and healthcare systems in tertiary hospitals, particularly among hospitalized patients. Multidrug resistance genes, including ESBLs, AmpC beta-lactamases, carbapenemases, and MBLs, significantly reduce the number of effective antibiotic treatment options [24]. In this study, overall, 52.8% (122 out of 231) of isolates were phenotypically ESBL-positive, and among these, 95.9% (117 out of 132) were confirmed to carry ESBL genes by PCR. This result is also consistent with other studies, showing up to 86.5% agreement in *Escherichia coli* ESBL-positive isolates [7]. The strong phenotypic–molecular agreement shows phenotypic testing is reliable, but combined methods are needed to detect a few undetected variants or other resistance mechanisms. Such mechanisms may include efflux pump overexpression, porin loss, or reduced membrane permeability, and target site alterations, none of which are detected by the PCR targets used in this study.

Out of 189 phenotypically confirmed DRM, 76.2% had PCR-confirmed drug resistance enzyme-encoding genes. This is comparable to the study done in Ethiopia, with 75% having drug-resistance genes [25], and lower than the study done in the Asian Pacific regions, 86.2% [26]. The number of non-DRM isolates increased from 42 by phenotypic confirmation to 55 PCR-negative GNB. Thirteen strains that were drug-resistant in the phenotyping confirmatory test were PCR-negative, possibly due to genes not included in this study or other than enzyme production DRM. Among PCR-confirmed beta-lactamase producers, ESBL, MBL, carbapenemase, and AmpC accounted for 53.2%, 21.6%, 19.9%, and 9.5%, respectively. Correlation analysis indicated a strong association between phenotypic and molecular characterization for ESBL, AmpC-BL, carbapenemase, and MBL.

The most commonly detected genes in this study were *blaCTX*-M (85.2%), followed by *blaTEM* (79.5%) and *blaBEL* (17.2%). The rapid spread of *blaCTX-M* is consistent with global trends, where *blaCTX-M*-type variants have largely replaced past *blaTEM* and *blaSHV* variants as the dominant, and this brings a change in ESBL epidemiology [27,28]. There is also a substantial proportion of isolates in this study that showed the co-occurrence of *blaCTX*-M and *blaTEM*, which may indicate horizontal gene transfer mediated by plasmids, transposons, and integrons, contributing to multidrug resistance [29]. Among minor ESBL genes, *blaBEL* 17.2% was more prevalent than the commonest *blaSHV* 9.8%. This suggests that drug-resistance gene prevalence and distribution fluctuated over time [20]. These findings underscore the dynamic nature of ESBL gene distribution and highlight the importance of continuous molecular surveillance to track resistance evolution.

In this study, *Klebsiella species*, particularly *Klebsiella pneumoniae,* were the predominant ESBL-producing bacteria, aligning with global trends where *Klebsiella pneumoniae* plays a key role in disseminating AMR genes from environmental microbes to clinically important pathogens [30]. The most commonly detected ESBL genes among *Klebsiella pneumoniae* in this study were *blaTEM* and *bla*CTX*-M*, each found in 51 of the isolates (87.9%), highlighting their co-dominance and significant contribution to β-lactam resistance. The high prevalence of these genes supports findings from other studies, which report that *blaTEM* is also present in a substantial proportion. These *blaTEM* and *blaCTX*-*M* genes were prevalent in *Escherichia coli*, which is widely recognized as a major carrier of ESBLs and widespread dissemination of variants in *Enterobacteriaceae* [31]. In contrast, *blaSHV* was less common, detected in only 13.8% (8/58) of the isolates. However, historically, *blaSHV* variants were among the earliest ESBLs identified in *Klebsiella* species. The continued dominance of *blaCTX*-M and *blaTEM* suggests ongoing horizontal gene transfer and the evolution of multidrug-resistant *Klebsiella pneumoniae* strains.

In this study, *blaCTX*-*M* was the most prevalent ESBL gene detected among *Escherichia* species, identified in 95.8% (23/24) of the isolates, and in hospital settings. The predominance of *blaCTX-M* is consistent with global epidemiological trends, where *blaCTX-M* enzymes have largely replaced older ESBL types as the dominant resistance mechanism in *Escherichia coli* [28]*.*

The AmpC genes confer resistance, and they can be easily disseminated by horizontal gene transfer [32]. In this study, 14.3% (33/231) of GNB isolates were phenotypically positive for AmpC production, reflecting the growing emergence of these resistant strains in clinical settings. Molecular confirmation via PCR revealed that 66.7% (22/33) of phenotypically positive isolates harbored AmpC genes, indicating a moderate concordance between phenotypic and genotypic methods. This discrepancy may be attributed to either non-AmpC resistance mechanisms or limitations in phenotypic assays, as supported by recent findings [32].

The *blaFOX* and *blaCITM* genes were the most frequently detected, accounting for 39.4% and 36.4%, respectively. The predominance of *blaFOX* and *blaCITM* is consistent with other regional and global reports that have documented its high prevalence [33,34]. The highest rate of AmpC positivity was identified in *Escherichia coli* (21.1%), with the *blaCITM* gene detected in 87.5% of these isolates, mirroring similar trends reported in other countries, where this gene has shown wide dissemination [34,35]. The *Klebsiella* Spp (10.1%), in which the *blaFOX* gene was the most frequently detected, were present in 54.5% of cases. This also accords with other studies [35,36]. The high prevalence of *blaFOX* and *blaCITM* might be due to their plasmid-mediated spread, strong resistance to cephalosporins, and association with *Klebsiella* spp. and *Escherichia coli*, respectively.

Pseudomonas spp were positive for AmpC confirmatory E-test and do not express AmpC-BLs genes. This resistance may be due to mechanisms other than hydrolytic enzyme production; for example, efflux pumps are common in non-fermenters, and alterations at antibiotic-binding sites could also contribute. In addition, ten bacterial species do not express the AmpC-BLs producer gene. Continued molecular surveillance is therefore essential to monitor resistance trends, identify emerging gene variants, and inform targeted therapeutic and preventive strategies.

In general, resistance to carbapenems may occur due to three major mechanisms, which include production of β-lactamase enzymes (carbapenemases), overexpression of efflux pumps, and porin-mediated resistance [37,38]. In this study, a total of 69 (29.9%) isolates were confirmed as carbapenemase producers by antimicrobial susceptibility and CIM testing. Carbapenemase producers were found to be higher compared with other studies conducted in Ethiopia (7.7%) [25] and 16% [39], 3.8% in Sudan [40], and 14.7% in the Asia-Pacific region [26]. Of the 69 isolates that showed positive phenotype, 63 (91.3%) were PCR confirmed to carry the gene encoding carbapenemase. The isolates that were PCR confirmed to carry the carbapenemase and Metallo-beta-lactamase encoding genes were 46 (66.6%) and 50 (72.5%), respectively. The findings highlight the aggressiveness and spreading of carbapenem resistance and the need for broader genetic screening and advanced tools like whole-genome sequencing for accurate detection.

The most common GNB with carbapenemase and MBL producing genes were *Acinetobacter* spp (42.6% and 46.8%) and *Klebsiella pneumoniae* (16.0% and 17.3%), respectively. The most predominant carbapenemase genes in *Acinetobacter spp* were *blaOXA23* (55.2%) and *blaOXA*-58 (13.8%), whereas in *Klebsiella pneumoniae, blaKPC* (33.3%) and *blaOXA23* (27.8%).

Among carbapenemase-encoding genes *blaOXA-23* 28(40.6%) and *blaKPC* 10(14.5%) was predominant. The higher *blaOXA-23* prevalence in Ethiopia is a new report compared with other African and Asian countries, except Taiwan, in which a similar finding was reported [26]. The reason could be that most Ethiopian patients went to Taiwan for medical tourism. The most prevalent MBL producer genes were *blaNDM* (39.1%) and *blaVIM* (34.8%). The highest prevalence of *blaNDM* in our study showed agreement with the study done in Taiwan [26]. The higher *blaNDM* was recorded in *Klebsiella pneumoniae* 13(72.2%) in this study. A study done in Addis Ababa TASH suggested carbapenem resistance to *Klebsiella pneumoniae* may be associated with medical tourism in Taiwan.

## Conclusion

The phenotyping and molecular characterization of DRM show a strong association. This study highlights a high prevalence of beta-lactamase-producing genes among Gram-negative bacteria, with ESBLs being the most dominant, particularly blaCTX-M and blaTEM. The emergence of blaBEL over blaSHV suggests evolving resistance patterns, while the detection of blaOXA-23 at high levels marks a significant and previously undocumented finding in Ethiopia. Ongoing molecular surveillance, improving laboratory capacity, updating treatment guidelines, and reinforcing antimicrobial stewardship and infection prevention measures are essential to reduce resistance and improve patient outcomes.

### Limitation

The limitation of this study is that some isolates showing phenotypic resistance were PCR-negative. This may occur because PCR detects only the specific genes included in the assay, whereas phenotypic AST reflects all resistance mechanisms, including those not gene-based or not covered by the PCR panel. Phenotypic detection of MBL resistance is lower compared to genotypic detection. This is because many strains carry both carbapenemase and metallo-β-lactamase resistance genes. For eCIM testing to be applicable, the isolate must be carbapenem-resistant, and the mCIM method test must be negative. However, most strains are positive for both mCIM and eCIM, limiting the ability to distinguish MBL producers phenotypically.

## Supporting information

S1 File Raw_images. (DOCX)

S2 File Table. (XLSX)

## Abbreviation:

AST: Antimicrobial susceptibility test
DRM: Drug resistance mechanism
ESΒL: Extended-spectrum beta-lactamase
AmpC-BL: AmpC-beta-lactamase
MBL: Metallo beta-lactamases
Carba: Carbapenemase
ICU: Intensive care unit
NICU: Neonatal ICU
SICU: Surgical ICU
MICU: Medical ICU
SWI: Surgical wound infection
RTI: Respiratory tract infection
UTI: Urinary tract infection
