# Supplementary material for: Resistance gene profiling of beta-lactamase and carbapenemase in gram-negative blood isolates: A tertiary care hospital
Source: PLoS One. 2026 Mar 30;21(3):e0344856. doi: 10.1371/journal.pone.0344856 (PMC13035159; doi:10.1371/journal.pone.0344856)
Supplement: S1 File — (DOCX) [file pone.0344856.s001.docx]

Supporting information: raw blot and uncropped full gel images from the gel documentation reading.

| **A** |
| --- |
| 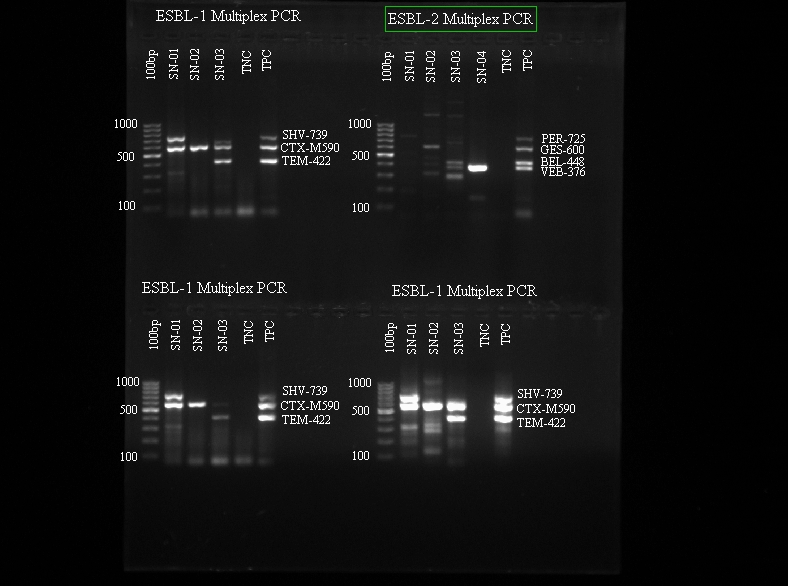 |
| **B** |
| 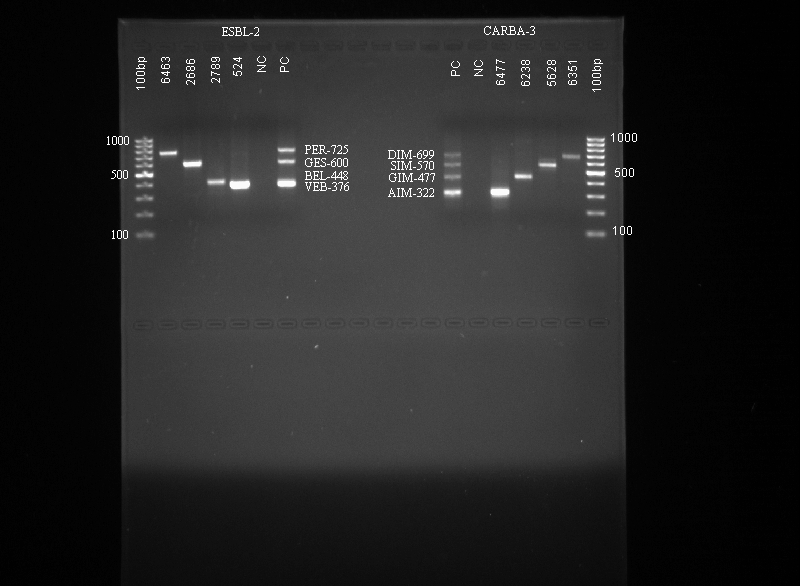 |

**S1_raw_image 1:** Multiplex PCR ESBL 1 and ESBL 2 full Gel doc reading. a). ESBL multiplex PCR 1, Major ESBL for the detection of *blaSHV, blaCTXM,* and *blaTEM* genes from 3 patient samples with TNC-negative and TPC-positive control. b). ESBL multiplex PCR 2, Minor ESBL for the detection of *blaPER, blaGES, blaBEL,* and *blaVEB* genes from 4 patient samples with NC-negative and PC-positive control.


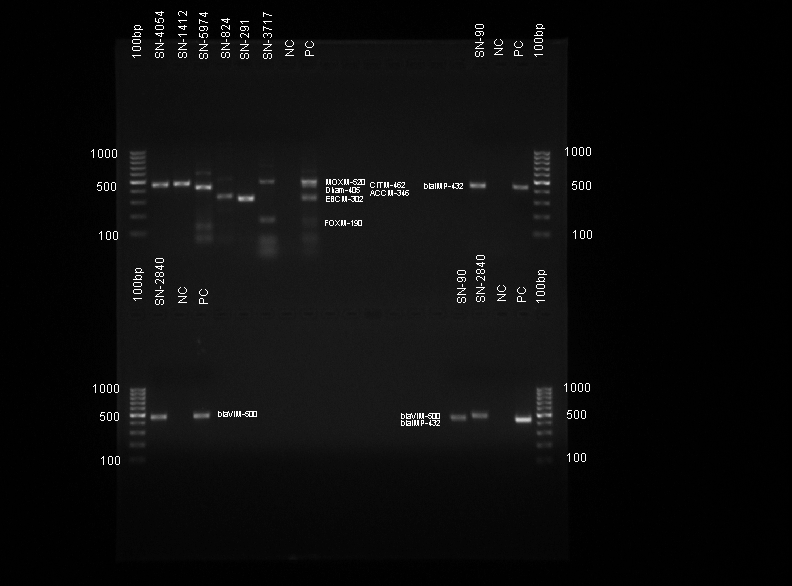
 S2_raw_image 2: Multiplex PCR ESBL and AmpC 3 Gel doc reading. ESBL Multiplex PCR 3, AmpC-BL gene detection of *blaMOXM, blaCITM, blaDHAM, blaACCM, blaEBCM,* and *blaFOXM* genes of 6 patient samples with NC-negative and PC-positive control

| **A** |
| --- |
| 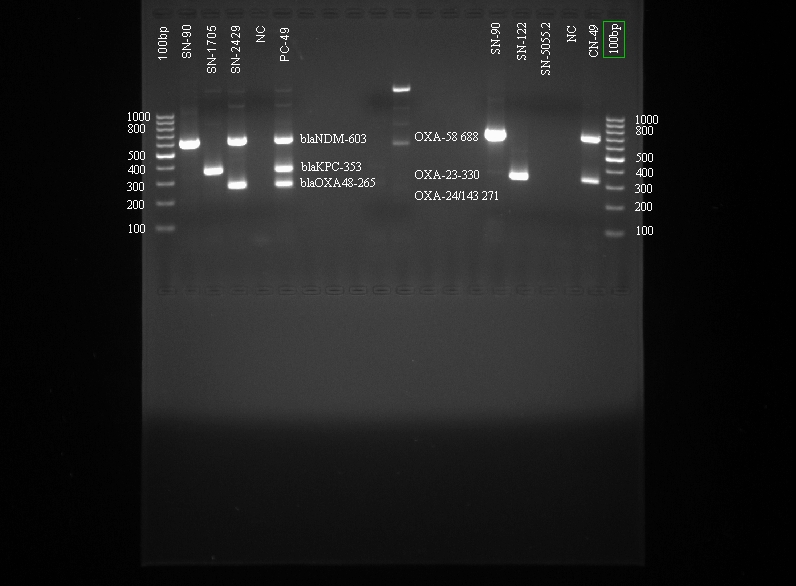 |
| **B** |
| 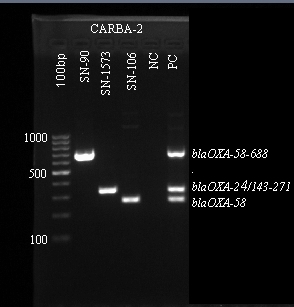 |

**S3_raw_image 3:** Multiplex PCR CARBA 1 and Multiplex PCR CARBA 2 Gel doc reading. a). CARBA Multiplex PCR 1, Carbapenemase gene detection of *blaNDM, blaKPC*, and *blaOXA-48,* genes of 3 patient samples with NC-negative and PC-positive control. b). CARBA Multiplex PCR 2, Oxacillinase gene detection of *blaOXA-58, blaOXA-23,* and *blaOXA24/143*, genes of 3 patient samples with NC-negative and PC-positive control.

| **A** | **B** |
| --- | --- |
| 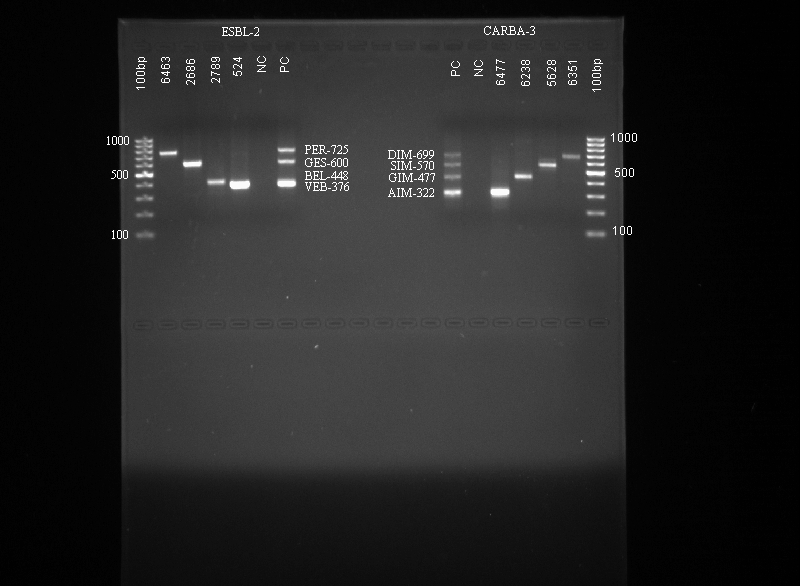 | 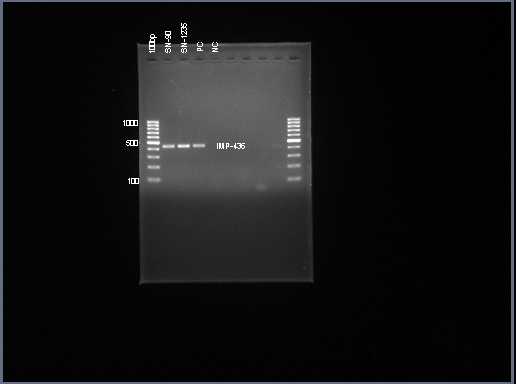 |
| **C** | |
| 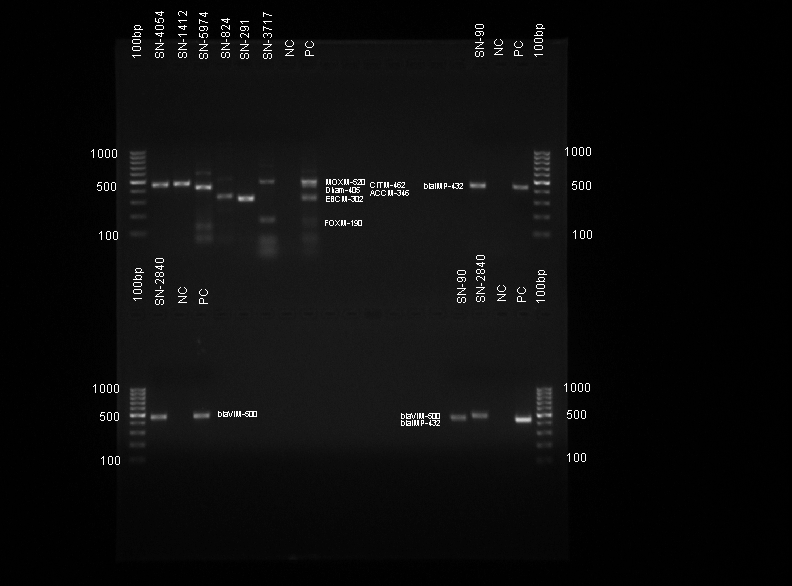 | |

**S4_raw_image 4**: Multiplex PCR CARBA 3 Gel doc reading. a). CARBA Multiplex PCR 3, Minor MBL gene detection of *blaDIM, blaSIM, blaGIM, and blaAIM* genes of 4 patient samples with NC-negative and PC-positive control. b). CARBA Multiplex PCR 1, MBL gene detection of *blaIMP* genes of patient samples with NC-negative and PC-positive control. c). CARBA Multiplex PCR 1, MBL gene detection of *blaVIM* genes of patient samples with NC-negative and PC-positive control.
